# Water source most suitable for rearing a sensitive malaria vector, *Anopheles funestus* in the laboratory

**DOI:** 10.12688/wellcomeopenres.12942.2

**Published:** 2018-01-29

**Authors:** Genevieve Tchigossou, Romaric Akoton, Akadiri Yessoufou, Innocent Djegbe, Francis Zeukeng, Seun M Atoyebi, Eric Tossou, Kabirou Moutairou, Rousseau Djouaka

**Affiliations:** 1 International Institute of Tropical Agriculture (IITA), Cotonou, Benin; 2 Laboratory of Cell Biology and Physiology, University of Abomey-Calavi, Abomey-Calavi, Benin; 3 University of Sciences, Arts and Techniques of Natitingou, Natitingou, Benin; 4 Faculty of Science, Department of Biochemistry, University of Yaounde I, Yaounde, Cameroon; 5 Cell Biology and Genetics Unit, Department of Zoology, University of Ibadan, Oyo State, Nigeria

**Keywords:** Anopheles funestus, rearing, eggs, larvae, F1 generation, borehole water, mineral water, physico-chemical parameters

## Abstract

**Background:**  The insecticide susceptibility status of *Anopheles funestus,* one of the main malaria vectors in the Afrotropical regions, remains under-studied due to the difficulty of working with this mosquito species. Collecting their larvae in natural breeding sites, rearing and maintaining them in normal laboratory conditions have been a difficult task. Forced-egg laying technique has been a very good tool to generate eggs from adult mosquitoes collected from the wild but rearing these eggs to obtain satisfying portion as adults has always been the problem. In this study, we optimized the development of mosquito species larvae under standard laboratory conditions for desired production of adult mosquitoes that can be useful for insecticide susceptibility tests.

**Methods:  **A forced-egg laying technique was used to obtain eggs from gravid female *Anopheles funestus* collected from Kpome locality in Benin. Eggs were reared in three different water samples (water from the borehole, and two mineral water namely FIFA and Possotômè) and larvae were fed with TetraMin baby fish food. The physico-chemical parameters of the waters were investigated prior to use for egg incubation (introduction of eggs’ batches into water).

**Results: **In contrast to mineral water that had no contamination, the borehole water source was contaminated with lead (2.5mg/L) and nitrate (118.8mg/L). Egg hatching rates ranged as 91.9 ± 4.4%, 89.1 ± 2.5% and 87.9 ± 2.6% in FIFA, Possotômè and borehole water respectively. High emergence of larvae to adult mosquitoes was recorded as in FIFA (74.3%) and Possotômè (79.5%) water. No adult mosquito was obtained from larvae reared in borehole water.

**Conclusions: **This study gave insight on the water sources that could be good for rearing to mass produce *An. funestus* in the laboratory. More analysis with other local mineral water sources in our environments could be considered in the future, hopefully giving better outputs.

## Introduction

*Anopheles funestus* remains a main malaria vector, and is thereby also responsible for malaria morbidity and mortality in Sub-Saharan Africa ^1^. Breeding of this mosquito species like other mosquitoes also requires an aquatic environment where larvae emerge to adult mosquitoes. Water is an important component of the ecosystem of this insect, and the quality of the water is an important determinant in egg laying, for adequate growth and development from larval stages until adults ^2– 5^. *An. funestus*, unlike the other known malaria vector, *An. gambiae,* in Sub-Saharan Africa breeds in natural/artificial permanent and semi-permanent water bodies with floating or emerging vegetation like edges of swamps, in weedy and grassy parts of rivers, streams, furrows, ditches and ponds with low salinity and little richness in organic matter ^6^. There are reports that *Anopheles* mosquitoes breed in clear waters with temperatures between 22°C and 32°C ^7^. However, decreased oxygen levels caused by water flow and flooding are always responsible for physical damage of mosquito larvae ^8^. In addition, breeding water with a pH range of 6.08 to 7.02 is good for weakening the egg shell, so that first instar larvae can emerge ^9^. Generally, these chemical properties of larval habitat, including ammonia, nitrate and sulphate concentration, influence larval development and their aquatic survival ^3^. Most experiments that study the biology of *Anopheles* mosquitoes, such as assessments of insecticide susceptibility, use laboratory reared colonies ^10^. It is therefore crucial to better understand suitable conditions for rearing of field collected mosquitoes. *An. funestus* is a difficult mosquito species to handle: not only because its larvae are rarely found during field survey, but also because of their inability to survive in normal laboratory conditions ^11^. Since *Anopheles funestus* represents an important malaria vector across Sub-Saharan Africa ^12– 15^, it is therefore necessary to find all means to study this malaria vector. Forced-egg laying technique has been a very helpful tool to generate a first filial generation of *An. funestus* mosquitoes from adults collected on the field ^16^. As much as this field tool has been of help, obtaining the desired quantity of *An. funestus* when rearing its larvae under laboratory conditions for insecticide susceptibility testing has been a big challenge. Sometimes, we recorded high mortality rate even when *An. funestus* mosquitoes are kept under recommended laboratory conditions. This observation prompted us to rear *An. funestus* larvae generated from forced-egg laying technique with different water sources but under the same laboratory conditions. Therefore, the aim of this study was to determine the most suitable water source which will obtain the best quantity of F _1_ *An. funestus* for laboratory experiments.

## Materials and methods

### Mosquito collection

Blood fed *An. funestus* resting indoors were collected in selected rooms at Kpome, a village (6°55′N, 2°19′E) located in the South of Benin. Collection was carried out between 06:00 and 10:00 am using electric aspirators. The collection period corresponds to the dry season in southern Benin when *An. funestus* densities are likely to be higher. Mosquitoes collected were morphologically identified as *An. funestus* group using the key of Gillies and Meillon. (1968) ^6^, kept in small cups and transferred to the laboratory (Insectariums of the International Institute of Tropical Agriculture of Benin) for rearing of the F _0_ to produce the F _1_ generation. .

### Mosquito rearing in the laboratory

**Figure 1.**
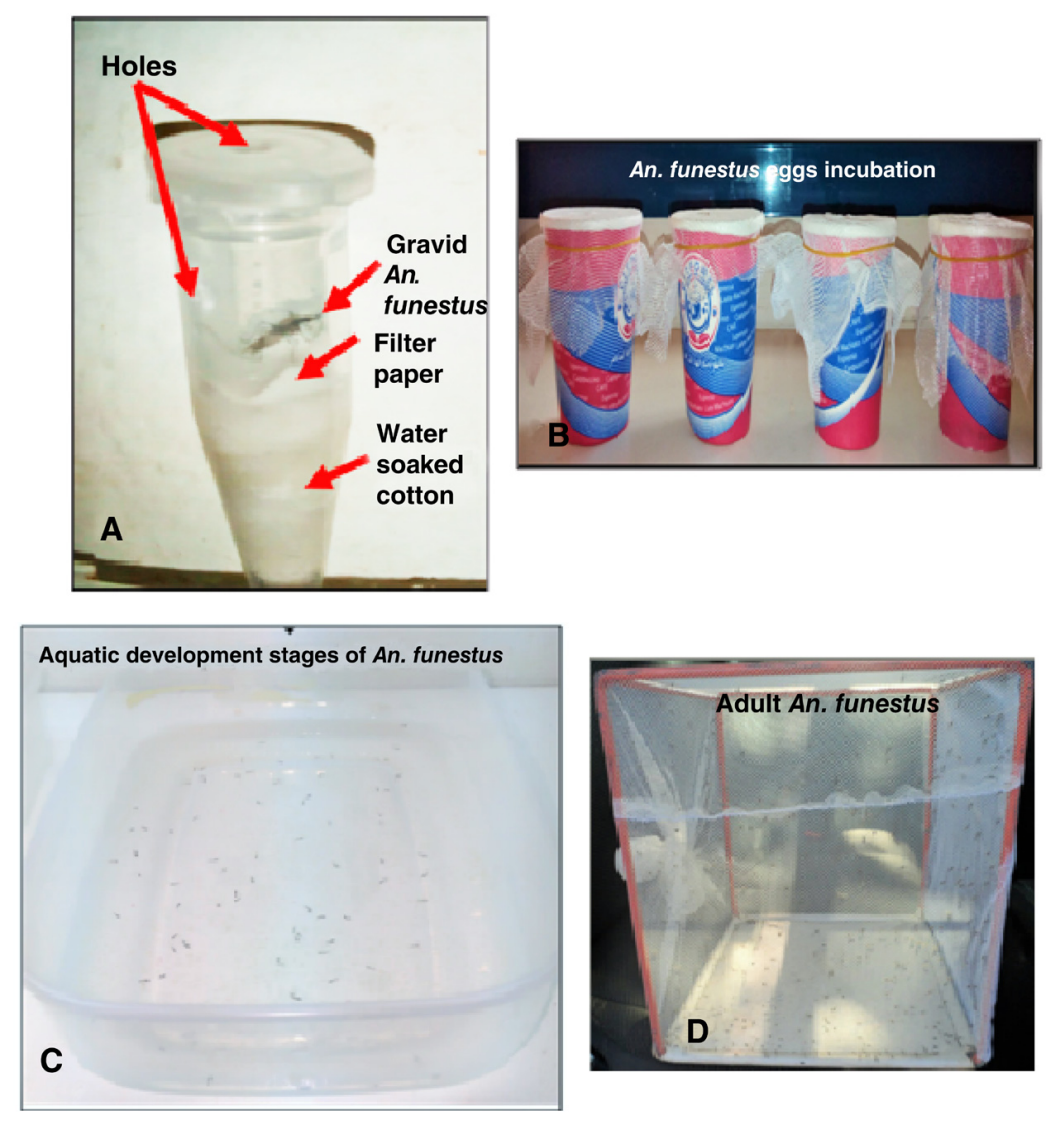
Developmental cycle of wild population of *An. funestus* in forced-eggs laying conditions: Oviposition of *An. funestus* ( **A**), Incubation and hatching of *An. funestus* eggs ( **B**), rearing of aquatic stage of *An. funestus* ( **C**), Adults emergence ( **D**).

In the insectary (T=25°C, RH=70–80% and L/D=12:12), blood fed, semi-gravid and gravid females were kept in the small paper cups (Diameter: 7.3 cm; height: 7.8 cm; capacity: 20cl) for 5 to 6 days after collection until fully gravid stage. Gravid females were then introduced individually and gently into 1.5ml Eppendorf tubes containing cotton soaked in water and surmounted by a filter paper (Wattman 3 mm/1 cm diameter) ( Figure 1) ^16^. Each Eppendorf tube was checked daily to identify females of *An. funestus* that have laid eggs, mosquitoes were gently removed from the tubes and transferred to a new Eppendorf tubes containing cotton and silica gel and stored at -20°C for subsequent experiments. Twenty-four hours post-oviposition, eggs from a single mosquito were divided into 3 batches and were allowed to hatch in small cups for larvae emergence, which were later transferred into rearing bowls containing 3 different types of water (Borehole water collected in Calavi, a southern Benin locality, and local mineral waters named FIFA and Possotômè) ( Figure 1). Water of each larvae bowl was changed every two days to reduce mortality and larvae were fed daily with Tetramin™ baby fish food. The larvae were monitored daily during the four larval stages of the mosquito up to the adult stage (F1 *An. funestus*). Each experiment was repeated at least 8 times with each type of water. The growth and development yield was evaluated based on:

(i) eggs hatching duration,
(ii) larvae rearing duration corresponding to duration from L1 stage till the first pupae stage,
(iii) larval mortality rate
(iv) adult emergence rate.

Sheets were established to collect data manually on these parameters, such as the number of eggs incubated in each type of water, the number of hatched eggs, larval and pupae mortality and number of daily emerged adult mosquitoes.

### Physico-chemical parameters of breeding water

 Each water sample was analyzed for physico-chemical parameters in the laboratory of water and food quality of Agriculture Environment and Health (AgroEcoHealth) platform of IITA-Benin. Temperature and pH of each water sample were determined using the pH meter WAG-WE30200 (Wagtech Projects, Berkshire, UK). Conductivity and Total dissolved solid (TDS) were also determined using the conductivity/TDS meter WAG-WE30210 (Wagtech Projects, Berkshire, UK). Before analysis, electrodes of these materials were sensitized, calibrated and rinsed with deionized water. Water samples were then analyzed, and all parameters were read and recorded. 

The quantity of nitrate, nitrite and chloride was determined using the Photometer 7100: WAG-WE10441 (Wagtech Projects, Berkshire, UK). Three replicates of each water sample were introduced into beakers and the reagents were added as recommended by the manufacturer (Wagtech Projects, Berkshire, UK). The mixture was incubated to stand for 10 min (Nitrate and nitrite) and for 2 min (chlorine) to allow the appearance of color. Each beaker was then inserted into the photometer and concentrations were directly displayed and recorded.

Calcium and fluorine were quantified using the W-22XD.23XD HORIBA multi-probe (HORIBA, ltd Japan). The electrodes were also calibrated and rinsed with sterile deionized water and then with the water samples to be analyzed. The quantities of calcium and fluorine were recorded after homogenization of the samples.

Heavy metals (Cadmium, Lead and Copper) were quantified using METALYSER HM 3000 (TRACE2O, Berkshire, UK) by the reverse voltammeter method. The electrodes were placed and conditioned according to the desired metal as recommended by the manufacturer. Heavy metal was quantified in 70 ml of water with appropriate reagents (buffer and standard) according to the desired metal. After 5 mins of incubation, the concentration of the metal and the corresponding graphs (in the form of a peak) were displayed on a tablet connected to the machine.

### PCR-based species identification

All females used for individual oviposition were identified using PCR as belonging to the *An. funestus* group. DNA was extracted from a total of 94 mosquitoes using the Livak protocol ^17^, followed by PCR species identification using the protocol described by Koekemoer *et al.* (2002) ^18^.

### Data analysis

Data were inserted on excel sheets and analyzed using SPSS 17.0. Chi square test was used to analyze the difference between hatching rate in different water samples. The easy to use online software (Fischer exact test) was used to assess the difference in larval mortality rate and adult emergence rate according to the water samples. The significance level was set at 5%.

### Ethical statement

The request for ethical approval was not applicable for this study, according to the International Institute of Tropical Agriculture (IITA) Ethical Committee (IITA, 08 P.O. Box 0932, Tri-Postal, Cotonou, Benin). However, there was a focus group discussion with the community and household heads where verbal and written consent was obtained for mosquito collections in the community after the study aims and objectives were explained. Since mosquitoes were collected using electrical aspirators, no insecticide spray or human bait methods were used for mosquito collections during this study.

## Results

### Species identification

Molecular identification of 94 females used for forced-eggs technique revealed that they all belonged to *An. funestus s.s*.

### Physico-chemical parameters of water samples

The physico-chemical parameters of the different water types used for *An. funestus* rearing are summarized in Table 1. These results showed that Possotômè mineral water had a pH of 7.9 whereas the pH obtained with FIFA mineral water and borehole water were 5.9 and 6.2, respectively. The total dissolved solids in Possotômè water (386 mg/l) was high compared to borehole water (131mg/l) and FIFA water (29.9mg/l). The same trend was observed with the total conductivity characterized by a high quantity of minerals in Possotômè water (775 μS/cm), which was significantly different to FIFA water (60 μS/cm) and borehole water (265 μS/cm) (p <0.05). Indeed, calcium and chloride concentration were higher in Possotômè mineral water (54 mg/l calcium, 110 mg/l chloride) than in FIFA mineral water (3.1 mg /l calcium, 10 mg/l chloride) and borehole water (0.00031 mg/l calcium, 12.2 mg/l chloride). Nitrate was almost absent in both mineral water (Possotome and FIFA) but was present at high concentration of 118.8mg/l in borehole water ( Table I). Lead was completely absent in mineral water while, it was detected in borehole water at a concentration of 2.5mg/l. Copper was also detected in all water samples (FIFA water: 0.00679 mg/l, Possotômè water: 0.042 mg/ml and Borehole water: 0.003399 mg/l) but at nontoxic-doses. No trace of cadmium was detected in all of water samples used for rearing. Temperatures recorded from pH meter were 25°C, 25.2°C and 25.5°C in FIFA, Possotômè and borehole water, respectively.

**Table 1.** Physico-chemical parameters of mineral/bottle waters (FIFA, Possotôme) and Borehole water.

Physico-chemical parameters	Borehole water	FIFA mineral water	Possotômè mineral water
pH	6.2	5.9	7.9
Total dissolved solids (mg/l)	131	29.9	386
Conductivity (μS/cm)	265	60	775
Calcium (mg/l)	0.00031	3.1	54
Chloride (mg/l)	12.2	10	110
Nitrate (mg/l)	118.8	4.04	0
Nitrite (mg/l)	0.0759	0	0.0462
Lead (mg/l)	2.58	0	0
Copper (mg/l)	0.003399	0.00679	0.042
Fluoride (mg/l)	0.38	0	0.3
Cadmium (mg/l)	0	0	0
Temperature (°C)	25.5	25	25.2

### Rearing yield of wild populations of *An. funestus*

A total of 355, 1124 and 830 *An. funestus* eggs were bred in borehole, FIFA and Possotômè mineral waters, respectively. There was no significant difference in the egg hatching duration between the borehole water (5 days) and mineral waters (4 days) (P = 0.0722). The eggs hatching rate of all repetitions (experiments) of each water sample are summarized in Table 2. These hatching rates varied between incubation days (the day the eggs were introduced in the water sample) and ranged from 12.61 ± 0.11% to 41.21 ± 6.11% for borehole, 16% to 55.57 ± 6.46% for FIFA and from 19.6 ± 2.38% and 43.77 ± 5.01% for Possotômè. No significant difference was found in the daily hatching rates for borehole (p = 0.0637), FIFA (p = 0.1450) and Possotômè waters (p = 0.080). Overall, no significant difference in eggs hatching rate was observed between the three waters (FIFA 91.9 ± 4.4%, Possotômè 89.1 ± 2.5% and borehole 87.9 ± 2, 6%) (P<0.05). Larval mortality rates obtained were respectively, 97.36%, 17.5% and 14.06% in borehole, Possotômè and FIFA waters ( Table 3). There was a significant difference in larval mortality with borehole water and mineral waters (p<0.05). No significant difference was observed in larval mortality between FIFA and Possotômè mineral waters (P = 0.3573). The percentage of adult mosquitoes that emerged from FIFA and Possotômè mineral waters were respectively 74.36% and 79.50%. No adult mosquitoes were obtained from borehole water ( Table 3). Another observation was that the rate of emerged adults in Possotômè was slightly higher than in FIFA mineral water but not significant (P = 0.1823). Rearing of *An. funestus* larvae to adults with both mineral waters took about 10 days, while for borehole water, rearing of larvae to pupae stage took as long as 15 days.

**Table 2.** Monitoring of emerged larvae of *Anopheles funestus* during the hatching period indifferent water samples.

Water samples	Number of replicates	Mean number of eggs per replicate	Eggshatchingdays	Meanhatching rate/day (%)	*p* value
***Borehole*** ***water***	8	44	1	0	0.0637
			2	41.21 ± 6.11	
			3	22.01 ± 4.82	
			4	12.07 ± 1.9	
			5	12.61 ± 0.11	
			**Total**	**87.9 ± 2.60**	
***FIFA mineral*** ***water***	15	75	1	0	0.1450
			2	55.57 ± 6.46	
			3	20.33 ± 11.54	
			4	16	
			**Total**	**91.9 ± 4.45**	
***Possotômè*** ***mineral water***	17	49	1	0	0.080
			2	43.77 ± 5.01	
			3	25.76 ± 2.63	
			4	19.6 ± 2.38	
			**Total**	89.13 ± 2.51	

**Table 3.** Adult productivity rate of *Anopheles funestus* rearing in different water samples.

Water samples	Borehole water	FIFA mineral water	Possotômè mineral water
**Number of replicates**	8	15	17
**Mean number of larvae/** **replicate**	38	64	40
**Mortality rates (%)**	97.36 (±5.08)	14.06 (±8.52)	17.5 (±11.78)
**Emerged adult rates (%)**	0	74.36 (±9.8)	79.5 (±11.3)
**larvae development** **duration (Days)**	15	10	10

## Discussion

Water quality is an important factor for oviposition of the female mosquito, and also influences the emergence of adult mosquitoes from larvae stages ^4^. However, physico-chemical parameters such as temperature, pH, dissolved oxygen, nitrate and sulphate concentrations are likely to affect the development and survival of mosquito larvae ^3^. The different values of physico-chemical parameters obtained in this study could give a better understanding on the environmental requirements needed to produce good yield of F _1_ *An.funestus* mosquito in the laboratory from the ones collected in the wild. The pH ranges (5.9 to 7.9) recorded in the different water sources could be considered suitable for mosquito breeding. These pH were similar to that found in breeding water samples, with pH values ranging from 4.0 to 7.8 considered favorable for normal development of *An. gambiae* under laboratory conditions ^9, 19^. pH values recorded in all sampling waters might not have an effect on the eventual yield of *An. funestus* in this study. A previous study also revealed that mosquito larvae can thrive well in water with neutral or slightly alkaline pH ^1, 20^. The temperature of water samples (25°C) used in this study is known to be suitable for *An. funestus* mosquito breeding ^7^.

This research also showed similar high hatching rates of eggs with FIFA, Possotômè and borehole waters, indicating that physico-chemical compositions of the different water samples do not influence the weakening of *An. funestus* egg shells. However, a high larval mortality rate (97%) was observed with borehole water compared to mineral water samples that produced good emergence rates. High larval mortality rate recorded could be attributed to the high nitrate dose in borehole water (118.8mg.l). This nitrate concentration in this water sample is higher than the maximum limit of 50mg/l nitrate dose authorized for human consumption ^21^, and could also be toxic for mosquito larvae. The main toxic action of nitrate on aquatic animals is the conversion of oxygen-bearing pigments (hemoglobin and hemocyanin) into the inhibited forms (methaemoglobin) which are not able to fix and carry oxygen ^22– 24^. Therefore, the lack of oxygen can cause the death of the mosquito larvae. The high nitrate content in the borehole water could be justified by the phreatic origin of this water, because ground water has always been described to contain toxic nitrate concentrations exceeding the standard ^9, 21^. Water sources that should be used for the breeding of aquatic stages of *An. funestus* should not have similar physico-parameters as ground water. Nitrate and phosphate residues derived from chemical fertilizers used in agriculture are other potential pollutants that could be found in aquatic waters ^25^. Ndenga *et al.*, (2012) ^26^ was able to establish a non-significant positive correlation between the presence of nitrate at 1.8 to 3.6 mg/l and the development of *Anopheles* larvae. It has also been demonstrated that the toxicity of nitrate decreased with increasing salinity of water ^27– 29^. This could further explain the high salinity of Possotômè mineral water, which had a high conductivity and high amount of total dissolved solids. Some studies have shown that at around 15.85 g/l of sodium chloride (NaCl) in water, salinity becomes a discriminate dose that kills *An. gambiae s.s*, *An. coluzzii* and *An. merus* larvae ^30^. It would always be good to assess any trace of phosphate and nitrate in rearing water before use for breeding *An. funestus* in the laboratory. However, the definite effect of nitrate on larvae development should be further investigated.

Despite a relatively high salinity of 110 mg/l in Possotômè water, more than 70% of adults were able to emerge. This correlates with the observation of Koekemoer *et al.*, (2014) ^31^, where they reported that salinity does not affect the emergence of *An. funestus* adults. Similar to egg hatching rates, rates of emergence of adult *An. funestus* in FIFA and Possotômè mineral waters were statistically similar, although the larval mortality rate was relatively low in FIFA water compared to Possotômè. No adult mosquitoes emerged from larvae reared with borehole water in this study. This could be explained by the fact that some larvae that reached pupal stage did not have enough energy to get out from their cuticle and become adults. This may be due to the lack of oxygen certainly related to high concentration of nitrate in borehole water ^22– 24^.

## Conclusions

This study highlighted the impact of some physico-chemical factors of breeding waters on *An. funestus* development under laboratory conditions. It showed that *An. funestus* could develop well in FIFA and Possotômè mineral waters, which both have similar physico-chemical characteristics. However, further studies should be performed to measure other physico- chemical parameters, such as phosphate, dissolved oxygen, alkaline content. This information will be of immense help to improve *An. funestus* rearing in order to obtain desired F _1_ progenies for more analysis. 

## Data availability

The raw data underlying the findings reported in this study can be found at: http://doi.org/10.17605/OSF.IO/AES4P ^32^.
