# Supplementary material for: Water source most suitable for rearing a sensitive malaria vector, Anopheles funestus in the laboratory
Source: Wellcome Open Res. 2018 Jan 29;2:109. Originally published 2017 Nov 10. [Version 2] doi: 10.12688/wellcomeopenres.12942.2 (PMC5721565; doi:10.12688/wellcomeopenres.12942.2)
Supplement: Peer review history

# Peer review: Referee response for version 1

**DOI:** 10.21956/wellcomeopenres.14033.r28752

**Published:** 2017-12-07

**Authors:** Emmanuel Elanga Ndille

**Affiliations:** 1 Research Institute for Development (IRD), MIVEGEC (Infectious Diseases and Vectors : Ecology, Genetics, Evolution and Control), Montpellier, France

This manuscript try to describe some qualities needed for water used for rearing *Anopheles funestus* mosquito species in the laboratory. The study deals with an important issue all medical entomologist faces to when trying to establish a lab stain of *Anopheles funestus* mosquito. Results obtained this manuscript of Tchigossou *et al* are very interesting and very informative. However even if the manuscript and data presented are interesting, there are some minor concerns I will like the authors to take into consideration so that their work will be more efficient.

**Comments**:

 1) In the section Material and methods, it would certainly be interesting for authors to give an approximate size of the small cup they used to keep their mosquitoes after identification. This aspect could be important as the manuscript try to present some interesting condition for the rearing of *Anopheles funestus* mosquito in the laboratory

 2) In the text (mosquito rearing in the laboratory) authors speak about “Calavi”. I think they should give more details on what is Calavi. Is not easy for the reader with no information about this locality to know what Calavi is.

 3- In the “mosquito rearing” section, it would have been interesting for authors to be precise that the total number of eggs reared was different or the same between the 3 types of water.

 4- As the present manuscript is trying to describe an experimental design to improve the rearing of *Anopheles funestus* in the laboratory, I think authors should ameliorate the quality of the pictures in figure 1. This will be helpful for someone who will try to reproduce what was done in the present study. Also it could be interesting for the reader if authors could give more details on “eggs incubation” 

 5- The two last sentences of the results section (Rearing yield of wild population of *An. funestus*) seem to be contradictory. Indeed, on one side authors said “No adult mosquitoes were recorded from borehole water”, on another side they said “… for borehole water, rearing of larvae to adult stage took as long as 15 days. It’s a bit difficult for the reader to understand how authors did to estimate these 15 days to obtain adult from borehole water, when it’s said by authors that with this water no adult mosquitoes were recorded? So maybe authors could clarify this last part of the “results” section.

I have read this submission. I believe that I have an appropriate level of expertise to confirm that it is of an acceptable scientific standard.

# Response

**Published:** 2018-01-14

**Authors:** Akadiri Yessoufou

**Affiliations:** University of Abomey, Calavi, Benin

**Answers, point by point, to the Reviewer’s comments**

The Reviewer approved our manuscript. However, he raised some minor points which we have now addressed and rectified accordingly in this revised version of the MS. The answers to his comments are as follows:

**Reviewer 1:** **Emmanuel Elanga Ndille**, Research Institute for Development (IRD), MIVEGEC (Infectious Diseases and Vectors: Ecology, Genetics, Evolution and Control), Montpellier, France 

1) We agree with the Reviewer’s comments. The characteristics of small cup used in this study were added in the revised manuscript. Please see in the *Material and Methods* section.

2)We agree with the Reviewer’s comment. More details have been given in the revised manuscript to explain what is Calavi. Please see in the *Material and Methods* section

3)We agree with the Reviewer’s comment. This precision has been given in the revised manuscript. Please see in the *Material and Methods* section

4) As per Reviewer’s suggestion, the quality of the pictures of figure 1 has been improved.

5) “eggs incubation” means introduction of the eggs batches into water. As per Reviewer’s suggestion, this precision has been given in the revised manuscript.

6) We agree with the Reviewer’s comment. Larvae reared in this water sample reached to pupae stage but no emergence was recorded. So, for this water sample “adult stage” was replaced by “pupae stage” in the revised manuscript. Please, see in the *Results* section.

# Peer review: Referee response for version 1

**DOI:** 10.21956/wellcomeopenres.14033.r27837

**Published:** 2017-12-01

**Authors:** Cyrille Ndo

**Affiliations:** 1 Malaria Research Laboratory, Organization of Coordination for the Fight against Endemic Diseases in Central Africa (OCEAC) , Yaoundé, Cameroon

**General comment**

 This is a very interesting paper, especially for all people working with *Anopheles funestus*. This species is difficult to colonize in insectary and this has for long time prevented research on *An. funestus*. By defining water conditions that could promote or impede larval development of this species, it will henceforth be easy to generate large F1 colonies from wild collected gravid females that could be used for various experiments.

**Recommendation**

 I recommend the paper to be accepted with minor revisions. Especially, the language in the abstract need to be improved.

**Reviewer's Comments and questions**

 Please check everywhere you have **bold** to see the change done

**Introduction**

 1) ... for adequate growth and **development from larval stages** until adults

 2) Insecticide susceptibility studies are important for checkmating the effectiveness of control tools used to control malaria vectors ^10^. Achieving success in this field requires a good understanding of the bio-ecology of the vector. This will guide us to organize suitable conditions to maintain mosquito samples collected from fields in the laboratory.

**Comment:** For me this transition is not appropriate. I suggest the authors to say rather that "most of experiments to study the biology of Anopheles mosquitoes, such assessment of insecticide susceptibility, use laboratory reared colonies. Is this therefore crucial to better understand suitable conditions  for rearing of field collected mosquitoes".

 3) Unfortunately, there are reports that *An. funestus *is involved in malaria transmission across Africa ^12 15^. It is therefore necessary to find all means to study this malaria vector.

**Comment:** I suggest the authors to say rather "Since Anopheles funestus represents an important malaria vector across Sub-Saharan Africa, it is therefore necessary to find all means to study this malaria vector."

 4) ........... for **insecticide **susceptibility testing.......

 5) Sometimes, we also have the problem of recording a high mortality and low hatching rate of field mosquitoes under suitable laboratory conditions because of their high sensitivity.

**Question:** This sentence is not clear. Can the author reformulate it?

 6) **Comment:**  I found the end of the introduction laborious from " Sometimes, we also have the problem..... " to the end

**Material and methods**

 7) larvae rearing duration corresponding to duration from oviposition till the first pupae stage

**Comment:** Most of the time, larvae rearing duration is the time from L1 to Puape

 8) **Comment:** Larval mortality, not larvae mortality

**Data analysis**

 9) Chi square test was performed to analyze the difference in hatching rate between different water samples and hatchingduration according to the water types.

**Comment:** I guess that hatching duration was expressed in days and that the authors rather compared means. I don't understand why the chi square test was used.

**Ethical statement**

 10) Mosquitoes were collected in the morning using electrical aspirators. No insecticide spray or human bait methods were used for mosquito collections during this study.

**Comment:**  I suggest the authors to say rather "Since mosquitoes were collected using electrical aspirators, no insecticide spray or human bait methods were used for mosquito collections during this study."

**Results**

 11) Nitrate was almost absent in both mineral water (Possotome and FIFA) but was present in high concentration of 118.8mg/l in borehole water (Table I).

**Comment**: Nitrate was almost absent in both mineral water (Possotome and FIFA) but was present **at** high concentration of 118.8mg/l in borehole water (Table I).

 12) **Comment and question:** I don't understand what the authors mean by egg hatching rate of all incubation days. Could they please explain or use a more clear expression?

 13) There was a significant difference in larvae mortality with borehole water and mineral waters (p<0.05).

**Comment:** The authors didn't mention if larval mortality was statistically different or not between the two mineral waters

 14) No adult mosquitoes were recorded from borehole water (Table 3).

**Comment:**No adult mosquitoes were **obtained** from borehole water (Table 3).

 15) Another observation was that the rate of emerged adults with Possotome was slightly higher when compared to FIFA mineral water.

**Comment:** Please add statistics

**Discusssion**

 16) These pH were similar to that **was** found in breeding water samples, with pH values ranging from 4.0 to 7.8 considered favorable for normal development of *An. gambiae *under laboratory conditions ^9,19.^

**Comment:** Remove "was"

 17) This research also showed **similarly** high hatching rates of eggs with FIFA, Possotome and borehole waters, indicating that physico- chemical compositions of the different water samples do not influence the weakening of *An. funestus *egg shells

**Comment:** Say rather "similar high..." or "comparable high...."

 18) **Question:** Can the authors use the term "larval mortality" rather than "larvae mortality"?

 19) This nitrate concentration in this water sample is higher than the maximum limit of 50mg/l nitrate dose authorized for human consumption ^21^ **, **and could also be toxic for the larvae development.

**Comment:** Say rather "This nitrate concentration in this water sample is higher than the maximum limit of 50mg/l nitrate dose authorized for human consumption ^21^ **, and could also be toxic mosquito larvae**.

I have read this submission. I believe that I have an appropriate level of expertise to confirm that it is of an acceptable scientific standard.

# Response

**Published:** 2018-01-14

**Authors:** Akadiri Yessoufou

**Affiliations:** University of Abomey, Calavi, Benin

**Answers, point by point, to the Reviewer’s comments**

The Reviewer approved our manuscript. However, he raised some minor points which we have now addressed and rectified accordingly in this revised version of the MS. The answers to his comments are as follows

**Reviewer 2: Cyrille Ndo**, Malaria Research Laboratory, Organization of Coordination for the Fight against Endemic Diseases in Central Africa (OCEAC) , Yaoundé, Cameroon 

**Introduction**

1)We agree with this correction and we have already incorporate the change in the revised manuscript. Please see in the Introduction section.

2)  As per Reviewer’s suggestion, we have now rectified the sentence accordingly. Please see in the *Introduction* section.

3) As per Reviewer’s suggestion, we have now reformulated the sentence. Please, see in the Introduction section 4) As per Reviewer’s suggestion **, **we have now introduced the word “insecticide” in appropriate place. Please see in the *Introduction* section.

5) As per Reviewer’s suggestion **, **we have now reworded the sentence. Please see last paragraph of introduction section: “Sometimes, we recorded high mortality rate even when *An. funestus* mosquitoes are kept under recommended laboratory conditions.”

6) As per Reviewer’s suggestion, we have now reformulated the last sentence of the introduction: “Sometimes, we recorded high mortality rate even when *An. funestus* mosquitoes are kept under recommended laboratory conditions. This observation prompted us to rear *An. funestus* larvae generated from forced-egg laying technique with different water sources but under the same laboratory conditions. Therefore, the aim of this study was to determine the most suitable water source which will permit to obtain the best quantity of F _1_ *An. funestus* for laboratory experiments.”

**Material and methods**

7) We agree with the Reviewer’s comment. “larvae rearing duration is the time from L1 to pupae”. We have now corrected this sentence in the revised manuscript.

8) We agree with the Reviewer’s comment. “larvae mortality” has been replaced by “larval mortality” throughout the revised manuscript.

**Data analysis**

9)We agree with the Reviewer’s comment. Chi square test was used to analyze the difference in hatching rate (means) between different water samples only not to compare duration (days). This statement has been corrected in the revised manuscript.

**Ethical statement**

10) As per Reviewer’s suggestion, we have now corrected the sentence. Please see in the *Material and Methods* section

**Results**

11) As per Reviewer’s suggestion, we have now corrected the sentence. Please see in the *Results* section

12) The tests have been repeated several times (see table 2: number of replicate). We have now corrected the sentence to better explain the idea: “The eggs hatching rate of all repetitions (experiments) of each water sample  are summarized in Table 2”

13) We agree with Reviewer’s comment. Larval mortality is not statistically different between the two mineral waters (P = 0.3573). We have now mentioned this in the revised manuscript.

14) As per Reviewer’s suggestion **, **we have now replaced “recorded” by “obtained” in the revised manuscript. Please see in the *Results* section

 15)  We agree with Reviewer’s comment. We have now corrected the sentence and added statistics to this statement in the revised manuscript (P = 0.1823).

**Discussion**

16) As per Reviewer’s suggestion, we have removed ‘was’ from the sentence in the revised manuscript. Please see first paragraph of the *Discussion *section

17) As per Reviewer’s suggestion **, **we have now corrected the sentence in the revised manuscript. Please see in the *Discussion* section

18) We agree with the Reviewer’s comment. “larvae mortality” has been replaced by “larval mortality” throughout the revised manuscript.

19) We agree with the Reviewer’s comment. The comment has been addressed in the manuscript and the sentence has been modified as suggested.
